# An immunoinformatics approach to design a multi-epitope vaccine against *Mycobacterium tuberculosis* exploiting secreted exosome proteins

**DOI:** 10.1038/s41598-021-93266-w

**Published:** 2021-07-05

**Authors:** Rahul Sharma, Vikrant Singh Rajput, Salma Jamal, Abhinav Grover, Sonam Grover

**Affiliations:** 1 Institute of Molecular Medicine, Jamia Hamdard, New Delhi, 110062 India; 2 School of Biotechnology, Jawaharlal Nehru University, New Delhi, 110067 India

**Keywords:** Computational biology and bioinformatics, Immunology, Diseases

## Abstract

Tuberculosis is one the oldest known affliction of mankind caused by the pathogen *Mycobacterium tuberculosis*. Till date, there is no absolute single treatment available to deal with the pathogen, which has acquired a great potential to develop drug resistance rapidly. BCG is the only anti-tuberculosis vaccine available till date which displays limited global efficacy due to genetic variation and concurrent pathogen infections. Extracellular vesicles or exosomes vesicle (EVs) lie at the frontier cellular talk between pathogen and the host, and therefore play a significant role in establishing pathogenesis. In the present study, an in-silico approach has been adopted to construct a multi-epitope vaccine from selected immunogenic EVs proteins to elicit a cellular as well as a humoral immune response. Our designed vaccine has wide population coverage and can effectively compensate for the genetic variation among different populations. For maximum efficacy and minimum adverse effects possibilities the antigenic, non-allergenic and non-toxic B-cell, HTL and CTL epitopes from experimentally proven EVs proteins were selected for the vaccine construct. TLR4 agonist RpfE served as an adjuvant for the vaccine construct. The vaccine construct structure was modelled, refined and docked on TLR4 immune receptor. The designed vaccine construct displayed safe usage and exhibits a high probability to elicit the critical immune regulators, like B cells, T-cells and memory cells as displayed by the in-silico immunization assays. Therefore, it can be further corroborated using in vitro and in vivo assays to fulfil the global need for a more efficacious anti-tuberculosis vaccine.

## Introduction

Tuberculosis (TB) disease is one of the oldest diseases of human mankind. It is an airborne communicable disease caused by the pathogen *Mycobacterium tuberculosis* (*M. tb*). The pathogen has coevolved with human from inception, since then we are living in the tuberculosis pandemic. According to the WHO Global Tuberculosis Report 2019, an estimate of 10 million people fell ill while 1.2 million died due to TB^1^. With the exceptional medical and science advancements, still we are struggling with tuberculosis treatment. Currently, available treatments for TB includes first-line drugs (isoniazid, rifampicin, ethambutol and pyrazinamide), second-line drugs (amikacin, kanamycin and capreomycin) and fluoroquinolones in combination with second-line of injectables^2^. The emergence of multi-drug resistance to TB due to the factors like improper treatment, poor life quality and limited supply of drugs have raised a great challenge against global TB management and prevention^3^. The only available TB vaccine BCG (Bacillus Calmette–Guérin) is given intra-dermally for pulmonary TB is struggling with its variable global efficacy, due to the reasons like the genetic variation in BCG strains, genetic variation among populations, interference by non-tuberculous mycobacteria (NTM) and interference by concurrent parasitic infections, thereby causing mortality and transmission^4,5^. To date, BCG vaccination in few countries is performed on a routine basis while few countries have adopted the targeted vaccination due to the reasons like BCG-associated deaths among immune-compromised children and a series of adverse events associated with BCG administration^6^.

In a biological system, cells communicate through the secretion of extracellular vesicle (EVs). EVs are cell membrane-derived lipid bilayer enclosing transmembrane proteins, cytosolic proteins and other biomolecules^7^. During the infection, EVs are released by the pathogen which contains the components of the pathogen including antigenic and immunogenic peptides, lipoarabinomannans (LAM and ManLAM) and pathogen-associated molecular patterns (PAMP’s)^8^. In recent years there are growing shreds of evidence supporting the role of EVs in pathogenesis^9,10^. Macrophages are the primary hosts which play a key role in *M. Tb* pathogenesis. Depending on the virulence of bacterial strain and the immediate environment of the infected macrophages *M. tb* may remain dormant for years or may get eliminated. Apart from the macrophages, neutrophils and the dendritic cell can also be infected by *M. tb* where the pathogen resides in the phagolysosome formed by the fusion of lysosome with the phagocytic vecuoles^11^. As evident from the literature, *M. tb* infected macrophages can traffic the bacterial molecules such as lipoarabinomannan (LAM and ManLAM) out of the phagolysosome by using the EVs^12^. Proteolysis of bacterial molecules by phagolysosome can generate peptides that can be antigenic or immunogenic and carried out by EVs.

In the present study, we have selected seven secreted mycobacterial proteins (DnaK, GrpE, LpqH, HBHA, LprA, LprG and MPT83) and a TLR4 agonist RpfE as vaccine adjuvant. All selected seven proteins are found to be present in the EVs of *M. tb* infected macrophages or of the *M. tb* itself^13^.

DnaK (Rv0350) is a conserved prokaryotic chaperonin protein that elicits innate and adaptive immune response through TLR2 and TLR4 immune receptors^14^. DnaK boosts the effectiveness of BCG vaccination proving its adjuvant like properties^15^. Intranasal immunization with DnaK displays similar protective effectiveness against *M. tb* as in the case of BCG vaccination^14^. GrpE (Rv0351) is a cofactor to the DnaK where both are encoded from a single operon^16^. GrpE also interacts with dendritic cells to elicit biased Th1-type T-cells immune response^17^. Both DnaK and GrpE are essential for the survival of *M. tb* which makes them promising targets for the development of tuberculosis treatment^18^. LpqH (Rv3763) is a 19 kDa mycobacterial lipoprotein that elicits T-cell proliferation and induces apoptosis in macrophages through the loss of mitochondrial trans-membrane potential in a caspase-dependent/independent manner^19^. Heparin-binding hemagglutinin abbreviated as HBHA (Rv0475) is a surface protein of *M. tb.* HBHA induces high secretion of IFN-γ in patients with latent tuberculosis infection (LTBI) and also triggers the extrapulmonary dissemination of the pathogen^20^. LprA (Rv1270c) is a mycobacterial cell wall-associated lipoprotein which can regulate the function of the antigen-presenting cells through an elevated level of various cytokines like TNF-α, IL-10 and IL-12 (34). LprG (Rv1411) is a mycobacterial lipoprotein which along with Rv1410c encodes for the membrane efflux pump, p55^21^. The O-mannosylated form of LprG bind to LAM, LM and triglycerides^22,23^. The LprG mutant show reduced exposition of LAM to the cell surface. *M. smegmatis* lacking LprG shows abnormal cell envelope morphology and permeability, indicating the role of LprG in the synthesis and maintenance of mycobacterial cell envelope^24^. MPT83 (Rv2873) is a secreted mycobacterial lipo-glycoprotein which is recognised by TLR2 and induces innate and adaptive immune response through the elevated level of cytokines like TNF-α, IL-6, IL-12 p40 and IFN-α^25^.

This study is an attempt to design an *in-silico* multi-epitope peptide vaccine combining the most potent antigenic, non-allergenic and non-toxic epitopes from the selected immunogenic proteins to respond to the global need for more efficacious TB vaccine. The protein sequences were evaluated to obtain the best possible B-cell, helper T lymphocytes (HTL) and cytotoxic T lymphocytes (CTL) epitopes using computational tools. All selected B-cell, HTL and CTL epitopes were screened for their antigenicity, allergenicity and toxicity before using them in vaccine construct. To ensure the wide coverage of the vaccine construct, selected CTL and HTL epitopes were also examined for their binding affinity for the most common human HLA alleles for MHC I and MHC II respectively. For better immunogenicity of the vaccine, a TLR4 agonist adjuvant peptide sequence was added to the vaccine construct. Multiple possible vaccine sequences were made and examined for their antigenicity, allergenicity, toxicity along with various physicochemical properties like the isoelectric point, solubility and half-life of the construct in various hosts. The homology modelling of all the vaccine sequences was performed and based on the online server’s recommendations and manual analysis, the best 3D model was selected. The stereochemical geometry of the 3D model was analysed for residue-by-residue geometry and overall structural geometry. Molecular docking of the 3D model with TLR4 was performed. The docked receptor-ligand complex was subjected to molecular dynamics simulation to confirm the stability of the docked complex in their natural biological environment. The final vaccine construct was codon optimised and *in-silico* cloned using an expression vector to ensure its expression. The vaccine construct was also subjected to immune simulation to validate our hypothesis.

## Methods and methodology

### Retrieval of bacterial proteins and TLR4 for vaccine construct

Amino acid sequences of DnaK (Rv0350, accession number-P9WMJ9.1), GrpE (Rv0351, accession number-P9WMT5.1), LpqH (Rv3763, accession number-P9WK61.1), HbhA (Rv0475, accession number-P9WIP9.1), LprA (Rv1270c, accession number-P9WK55.1), LprG (Rv1411c, accession number-P9WK45.1) And Mpt83 (Rv2873, accession number-P9WNF3.1) Along with the TLR4 agonist adjuvant RpfE (Rv2450c, accession number-CCP45243.1) were retrieved in fasta format from NCBI (National Centre of Biotechnology Information) protein database^26^. To select the best adjuvant for our vaccine construct, four literature approved TLR4 agonist proteins viz. 50 s ribosomal protein L7/L12 (Rv0652), CobT (Rv2207), HbhA (Rv0475) and RpfE (Rv2450c) were evaluated for their antigenicity in combination with the vaccine construct^27–29^. Both B-cell and T-cell epitopes were used for better innate and adaptive immune response.

### B-cell epitopes prediction

Linear B-cell epitopes were predicted using the ABCpred. The ABCpred is an online server that predicts linear B-cell epitopes in an antigenic protein using artificial machine learning^30^. The amino acid sequences of the selected proteins were submitted in a single letter code without any header line with 0.51 threshold individually, while epitopes length was selected as 16 mer with overlapping filter kept on. The resulting top five epitopes were selected for further studies.

### HTL epitopes prediction

HTL epitopes were predicted using the IEDB MHCII server. Immune Epitope Database and Analysis Resources (IEDB) is an online database and suite to predict immune epitopes from an extensive collection of experimentally derived immune epitopes^31^. The amino acid sequences of the selected proteins were submitted to the server independently in fasta format. The default (IEDB recommended 2.22) setting was selected for predicting the epitopes utilizing the full HLA human reference set. The epitope length was specified to be15 mer and results were sorted according to their adjusted ranks.

### CTL epitopes prediction

The CTL epitopes were predicted using the IEDB MHCI server. The amino acid sequence of the selected proteins was submitted in fasta format. The default (IEDB recommended 2020.04 (NetMHCpan EL4.0) setting was selected for predicting a 9 and 10 mer epitope while exploiting the complete human HLA reference set. The results were sorted according to the peptide score. Repeated HTL and CTL epitopes against different HLA alleles were removed from the study. Selected epitopes were examined further for their antigenicity, allergenicity and toxicity.

### Prediction of epitope’s antigenicity, allergenicity and toxicity

Before constructing the vaccine candidate all selected epitopes were subjected to their prediction for antigenicity, allergenicity and toxicity. Antigenicity was predicted using the VaxiJen online web server. VaxiJen can predict the antigenicity of a protein in an alignment-independent fashion based on its physicochemical properties^32^. Allergenicity was predicted using the AllerTop V. 2.0 web server. The AllerTop V. 2.0 uses an auto cross-covariance (ACC) approach where amino acid hydrophobicity, molecular size, helix-forming propensity, the relative abundance of amino acids, and β-strand forming propensity is used to calculate the allergenicity of the protein^33^. The toxicity was predicted using the ToxinPred server. It generates all possible mutants of given sequences and measures the toxicity for all of them along with their physicochemical properties such as hydrophobicity, pI, charges etc^34^. The VaxiJen server provides varied options of selecting the organism, for which it automatically specifies a threshold score. Bacteria and its concerned threshold score (0.4) were selected. For AllerTop V. 2.0 and ToxinPred all parameters were designated as the default. Only the epitopes which were antigenic, non-allergenic and non-toxic were further exploited for vaccine construction.

### Construction of subunit vaccine

Five random vaccine sequences were generated by changing the positions of the highest-scoring antigenic, non-allergenic and non-toxic B-cell, HTL and CTL epitopes. The B-cell, HTL and CTL were connected through KK, GPGPG and AAY linkers respectively. The amino acid sequence of RpfE adjuvant was linked to the N-terminal of all the vaccine constructs separately, using EAAAK linker. To select the best vaccine candidate, all five the vaccine constructs were evaluated for their antigenic properties using VaxiJen and AntigenPro. While the allergenicity of the construct was checked using AllerTop V. 2.0. Since all five vaccine constructs showed almost similar antigenicity and all were non-allergens, so we carried forward all the five vaccine constructs further into the study.

### Homology modelling of vaccine constructs

To select the best vaccine construct which shows stable protein structure all the five vaccine constructs were subjected for their homology modelling using the web servers like I-TASSER^35^, SWISS-MODEL^36^ and PHYRE2^37^. All generated models were downloaded in PDB format for further evaluations.

### Vaccine constructs tertiary structure validation

Generated tertiary structures of all five vaccine constructs were subjected for their validation using the Protein Structure Validation Server PSVS^38^. PSVS is a web server that evaluates the quality of the protein tertiary structure based on several parameters like pack and folds, local residue separations, deviation in bond angle and bond length, atomic overlaps, backbone and side-chain torsion angle. Based on the PSVS recommendations the best vaccine construct was selected for further structure refinement.

### Vaccine construct tertiary structure refinement

Refinement of the tertiary structure of the vaccine construct was carried out using the GalaxyRefine web server. GalaxyRefine uses molecular dynamic simulation for protein tertiary structure relaxation and structural perturbation. GalaxyRefine generates 5 refined models, for model 1 structure perturbation is applied to the only cluster of side chain while for models 2–5 more aggressive perturbation is applied where secondary structure and loops are also added. Results of GalaxyRefine were analysed for various parameters such as GDT-HA, RMSD, MolProbity, and clash score. The best-selected model was utilized for physicochemical properties assessment, molecular docking and dynamic simulations^40^.

### Vaccine construct physicochemical properties

The physicochemical properties of the vaccine construct were computed using the web server ProtParam tool^39^. ProtParam calculates the protein physicochemical properties on parameters like protein length, isoelectric point, molecular weight, half-life, instability index, aliphatic index and Grand Average of Hydrophobicity (GRAVY).

### Molecular docking of vaccine construct with human TLR4 receptor

The crystal structure of the human TLR4 (PDB ID-3FXI) complex was obtained from the protein database^41,42^. Molecular docking of the refined vaccine construct was performed with immune receptor human TLR4 in its monomeric form. The crystal structure complex of human TLR4 consists of 10 chains (A-H) where A and B chains are the human TLR4 receptor, C and D chains are lymphocyte antigen 96 while remaining E–H chains are different forms of glucopyranose^42^. The crystal structure complex of human TLR4 was prepared using the AutoDock Vina where the A chain of the structure was selected for docking and the rest of the non TLR4 molecules were removed from the complex^43^. Active pockets and ligand binding sites of the human TLR4 receptor and refined vaccine construct were determined using the CastP web server^44^. Molecular docking of the vaccine construct and the immune receptor human TLR4 was performed using the HADDOCK2.2 (High Ambiguity Driven protein–protein DOCKing) web server^45^. HADDOCK2.2 web server distinguishes itself from other docking servers in the fact that rather than performing the docking ab-initio, this server uses the data from identified protein interfaces to perform docking. On to the HADDOCK2.2 web server, the structure of both vaccine construct and immune receptor, human TLR4 was provided in PDB format along with their active site residues.

### Molecular dynamic simulation of the ligand-receptor complex

To conclude the stability of the docked complex in their natural biological environment, the vaccine-TLR4 complex was subjected to molecular dynamic simulation (MDS) using the GROMACS 5.0^46^. The MD simulation were performed for various parameters like RMSD (Root Mean Square Deviation), RMSF (Root Mean Square Fluctuation), Rg (Radius of gyration) and SASA (Solvent Accessible Surface Area). RMSD computes the simulation convergence using g_rmsd. RMSF was computed using the g_rmsf to study the deviation in the position of atoms. The radius of gyration (Rg) was calculated using the g_gyrate to compute the protein folding and compactness. SASA was performed using g_sasa to study the Area of protein exposed to the surface.MD simulation of the selected vaccine-TLR4 complex was performed for 50 ns to study the bonded and non-bonded characterization.

### Codon optimization and in-silico cloning of designed vaccine construct

To express the vaccine construct in *E. coli,* reverse translation and codon optimization of the designed vaccine construct were performed using JCat (Java Codon Optimization Tool) online web server. JCat results include CAI (Codon Adaptation Index) and GC content of the sequence^47^. The CAI value indicates the level of protein expression and a CAI value of > 0.8 is considered good^48^. The GC content of the sequence should be between 30 and 70%, while a GC content value out of this range results in reduced transcription and translation efficiencies^49^. The JCat input was submitted in amino acid sequence form and the host was selected as *E. coli* (strain K12) rest was set as default. To express the codon-optimized gene inside *E. coli, in-silico* cloning was carried out using the SnapGene tool. The BamHI and NdeI restriction sites were added to the N-terminal and C-terminal of the optimized gene respectively. The gene fragment with restriction sites was inserted inside the pET-28a(+) vector.

### Immune simulations

To interpret the immunogenicity and immune response profile of our vaccine construct, dynamic immune response simulations of the selected vaccine construct were performed using the C-ImmSim web server. The C-ImmSim is an agent-based immune simulator that uses Position-Specific Scoring Metrix (PSSM) to simulate the immune response. It consists of a dataset of 6533 antigenic epitopes and 33 different sets of human HLA alleles. C-ImmSim uses the amino acid sequence of antigenic epitopes in combination with lymphocyte receptors to simulate the immune response^50^. The vaccine construct was submitted to the C-ImmSim web server while keeping all the parameters to default.

## Results

### B-cell epitopes prediction

The top five epitopes predicted from the ABCpreds web server were selected for further analysis of their antigenicity, allergenicity and toxicity. Repeated epitopes selected against different HLA sets were removed from the study. The non-antigenic, allergenic and toxic epitopes predicted by VaxiJen, AllerTop V 2.0 and ToxinPred respectively were also removed from the study. In total sixteen B-cell epitopes were finalized for selected seven proteins (supplementary table1).

### HTL epitopes prediction

The top ten epitopes with antigenic, non-allergenic and non-toxic behaviour were selected for the vaccine construct. Repeated epitopes against different HLA sets were removed for the study. Nine HTL epitopes were finalized for five proteins except for GrpE and HbhA as no epitope for these two proteins qualified our criteria of antigenicity, allergenicity and toxicity (supplementary table1).

### CTL epitopes prediction

The top ten epitopes with the highest affinity to MHC I were shortlisted for their antigenicity, allergenicity and toxicity analysis. Repeated CTL epitopes against different HLA sets were removed from the study. The antigenic, non-allergenic and non-toxic epitopes were selected for further studies. Finally, ten CTL epitopes were selected for DnaK, LpqH, HbhA and LprA while no candidate epitopes for GrpE, LprG and Mpt83 qualified our criteria for short listing (supplementary table1).

### Vaccine construct preparation

Five random vaccine constructs were prepared by shuffling the positions of selected epitopes. Epitopes in vaccine construct were positioned as B-cell, HTL followed by CTL. The B-cell, HTL and CTL epitopes were connected through KK, GPGPG and AAY linkers. The RpfE protein sequence which served as an adjuvant was linked to the N-terminal of the vaccine construct through the EAAAK linker. All five sequences were subjected to their antigenicity and allergenicity prediction. All the vaccine constructs were found to be of antigenic and non-allergenic behaviour.

### Homology modelling and selection of best vaccine construct

**Figure 1:**
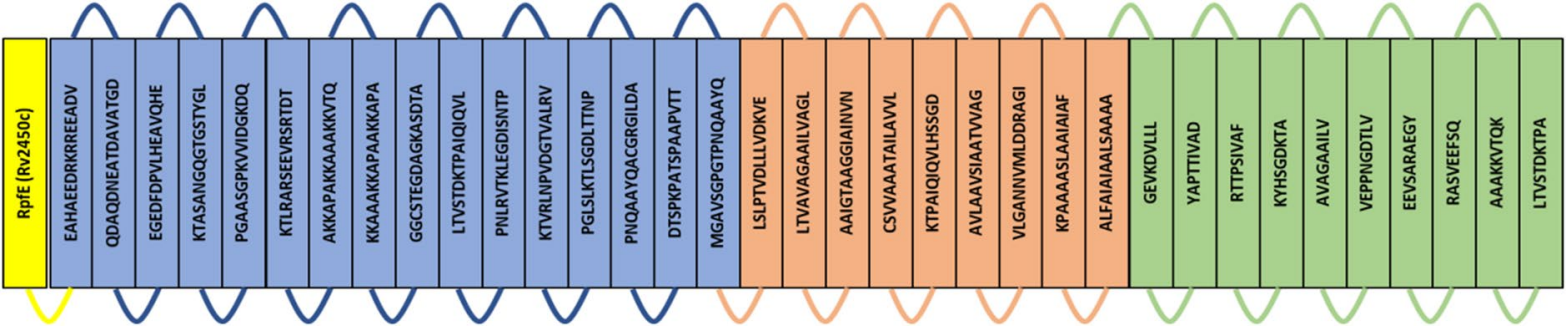
Diagrammatic representation of the final vaccine construct sequence where the sequence in boxes represents epitopes and connected through turns. The 766 amino acid long vaccine construct consists of 172 amino acids long adjuvant RpfE/Rv2450c (yellow), 16 B-cell epitopes (blue), 9 HTL epitopes (orange) and 10 CTL epitopes (green). Adjuvant RpfE connected to the construct at its N-terminal through EAAAK linker (yellow), B-cell epitopes are connected through KK linkers (blue), HTL epitopes connected through GPGPG linker (orange) and CTL epitopes connected through AAY linkers (green).

Homology modelled vaccine constructs were subjected to their structure validation using the PSVS server. PSVS reports of all constructs were analysed for their Ramachandran plot summary, mean score and Z-score. Vaccine construct sequence 1 modelled with PHYRE 2 web server showed the best results where values for Ramachandran most favoured regions, additionally allowed regions, generously allowed regions and disallowed regions were 91.5%, 8.5%, 0.0% and 0.0% respectively. For vaccine construct sequence 1, the mean values for Verify3D, ProsaII (−ve), Procheck G-factor ^e^ (phi/psi only), Procheck G-factor ^e^ (all dihedral angles) and MolProbity and clashscore were 0.31, 0.78, 0.17, 0.29 and 15.92 respectively. While the Z-score for Verify3D, ProsaII (-ve), Procheck G-factor ^e^ (phi/psi only), Procheck G-factor ^e^ (all dihedral angles) and MolProbity clashscore were -2.41, 0.54, 0.98, 1.71 and -1.21 respectively. The selected final vaccine construct was also subjected to antigenicity prediction using VaxiJen and AntigenPro web servers where antigenicity was predicted to be 0.95 from both web servers which is quite higher than the recommended threshold. The selected vaccine construct sequence is shown in supplementary Fig. 1 and the graphical representation of vaccine construct in order of epitopes utilized can be found in Fig. 1.

### Vaccine construct tertiary structure refinement

**Figure 2:**
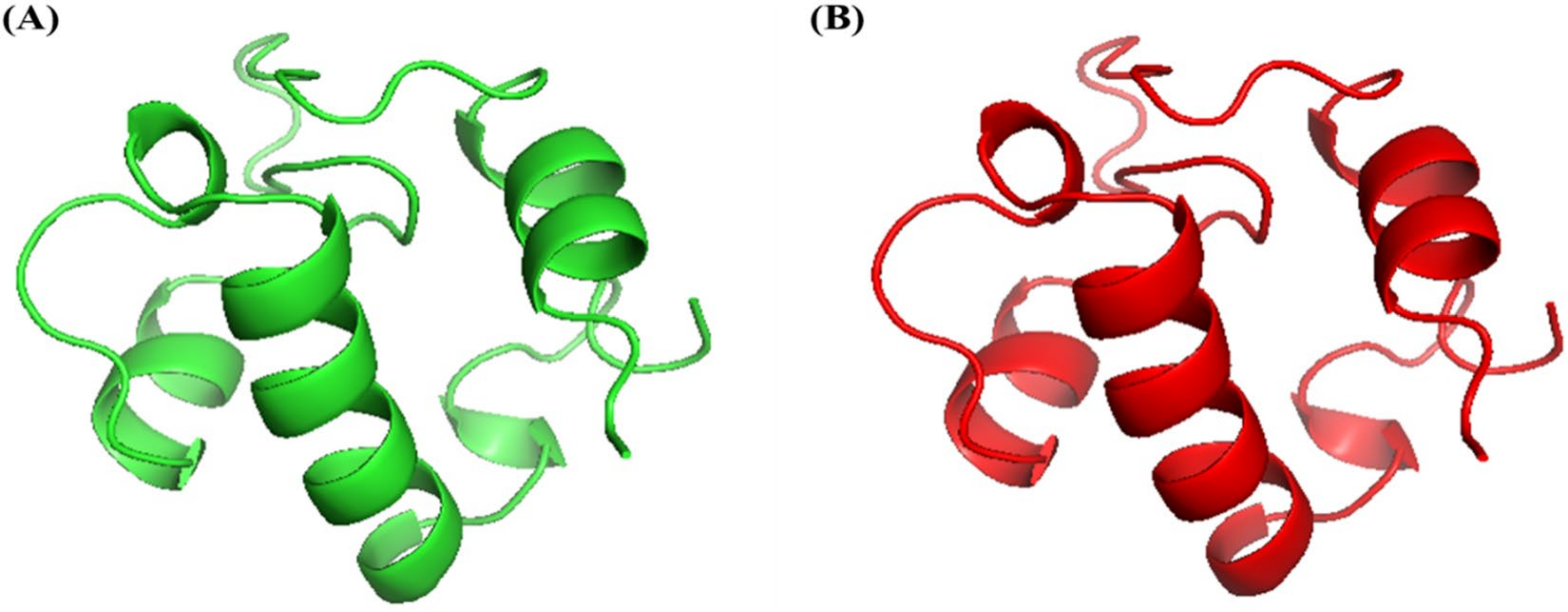
Vaccine construct homology modelling and structural refinement. (**A**) 3D structure of the vaccine construct was derived from the PHYRE 2 web server (http://www.sbg.bio.ic.ac.uk/phyre2). (**B**) The refined vaccine construct 3D structure was generated using theGalaxyRefine web server (http://galaxy.seoklab.org/refine).

**Figure 3:**
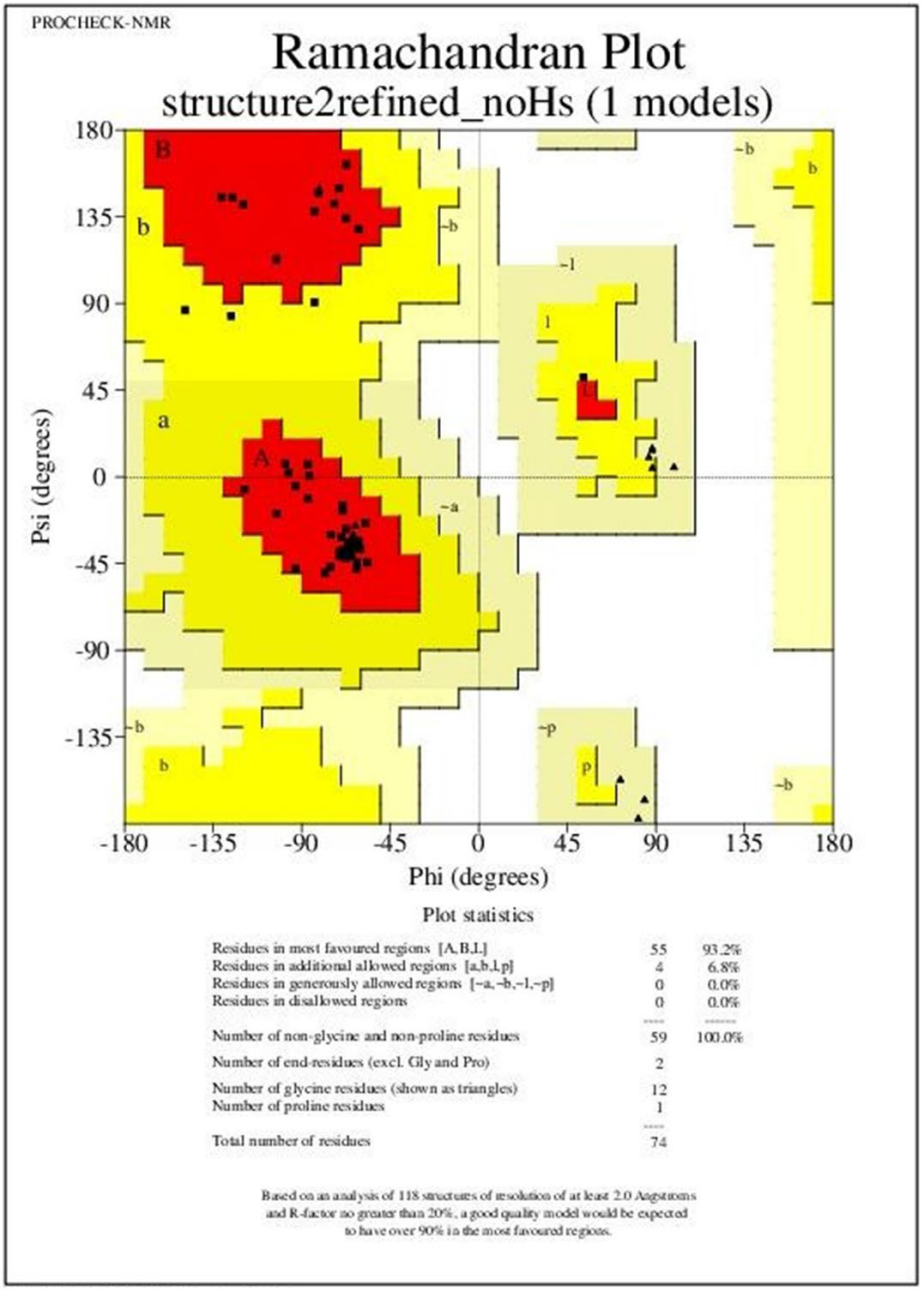
Ramachandran plot analysis of the refined tertiary structure of vaccine construct obtained from PSVS web server (https://montelionelab.chem.rpi.edu/PSVS/). After refinement most, favoured regions increased to 93.2% as compared to the crude 3D structure derived fromPHYRE2 (http://www.sbg.bio.ic.ac.uk/phyre2) where the most favoured regions were 91.5%.

The homology model generated using PHYRE 2 of vaccine construct sequence 1 (Fig. 2A) displaying the best Ramachandran plot values was subjected to refinement using GalaxyRefine. From the five models generated by GalaxyRefine, model 2 (Fig. 2B) was found to be most suitable where values for GDT-HA, RMSD and MolProbity were 1.0, 0.206 and 1.552 respectively. After refinement, the values of Ramachandran most allowed regions increased to 93.2% as compared to the crude structure derived from PHYRE2 (Fig. 3). Based on the provided results, the refined model 2 was selected for molecular docking and dynamic simulations investigations.

### Vaccine construct physicochemical properties

The length of the refined model 2 sequence selected as vaccine candidate was evaluated to be 766 amino acids with a molecular weight of 76.85 kilo Dalton. The peptide vaccine with molecular mass less than 50 kilos Dalton shows lower lymph node accumulation so, it’s better to have vaccine with a molecular mass higher than 50 kilos Dalton^51^. The theoretical isoelectric point of the construct was calculated to be 9.32 with 70 negatively charged residues and 89 positively charged residues which indicate that the constructed protein is basic. The estimated half-life of the construct in mammalian reticulocyte (in vitro) was 30 h while in yeast and *E. coli* the half-life was > 20 h and > 10 h respectively. The instability index was calculated to be 28.23 while an aliphatic index of the construct was 78.72 which suggest that the protein is thermally stable. The grand average of hydropathicity (GRAVY) of the construct was found to be -0.208.

### Molecular docking of vaccine construct and TLR4

**Figure 4:**
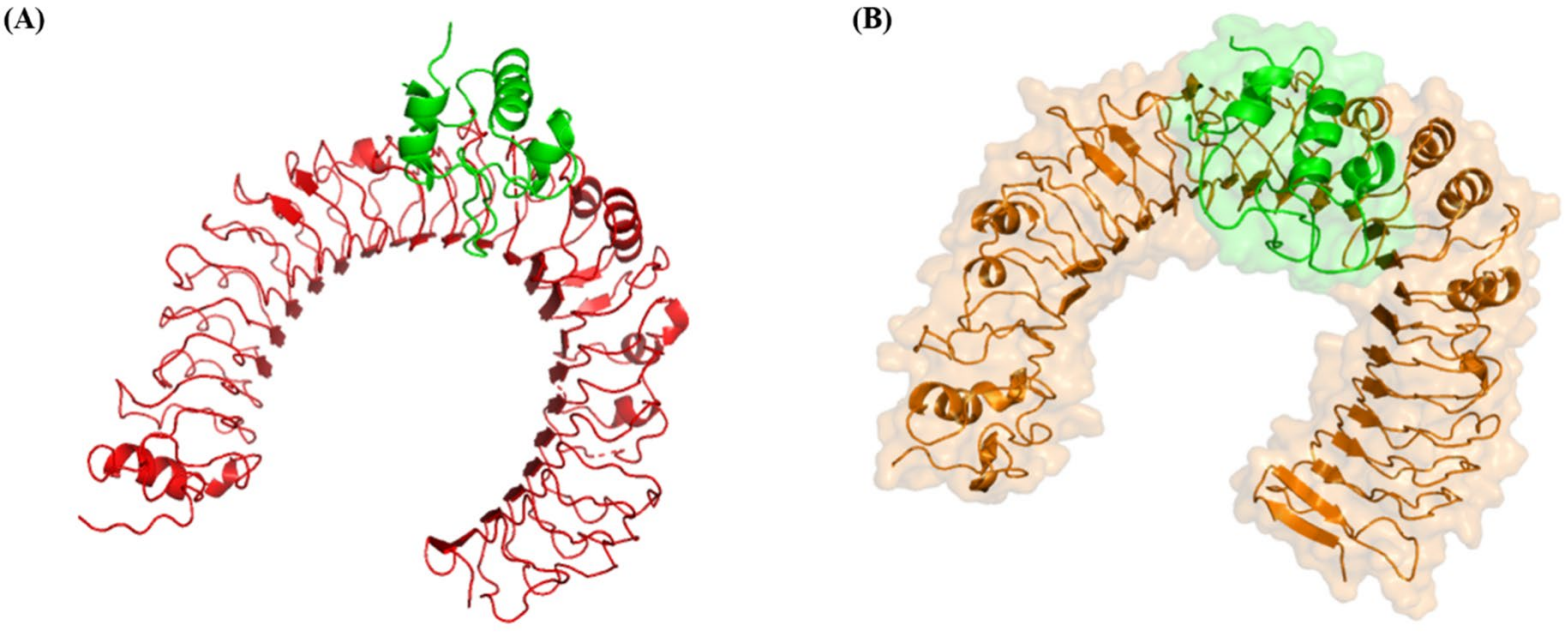
Molecular docking of vaccine construct and TLR4 immune receptor. (**A**) Molecular docking of the vaccine construct (green) with TLR4 immune receptor (red) obtained using HADDOCK web server (https://wenmr.science.uu.nl/). (**B**) Post MD simulation vaccine construct (green) complexed with TLR4 immune receptor (gold).

The CastP server was used to predict the active ligand binding sites in the refined tertiary vaccine structure. It predicted 9 (121, 126, 127,128, 131, 156, 160, 160, 164 and 165) residues as active ligand-binding sites for TLR4. The active pocket surface area and active pocket surface area volume of the vaccine construct were calculated as 42.24 Å and 24.74 Å respectively. The refined vaccine construct and prepared TLR4 monomer were submitted to HADDOCK2.2 web server along with their active ligand binding sites obtained from CastP web server. HADDOCK2.2 provided results in the form of 28 models in 7 clusters where each cluster contained 4 best-docked structures. The model 1 of cluster 1 displayed the lowest Z-score (− 1.2) among all 28 models predicted. Other calculated parameters are provided in supplementary table2. This selected vaccine-TLR4 complex was further analysed for the MD simulation. The docked vaccine construct and TLR4 receptor can be seen in Fig. 4A.

### Vaccine-TLR4 complex MD simulations

**Figure 5:**
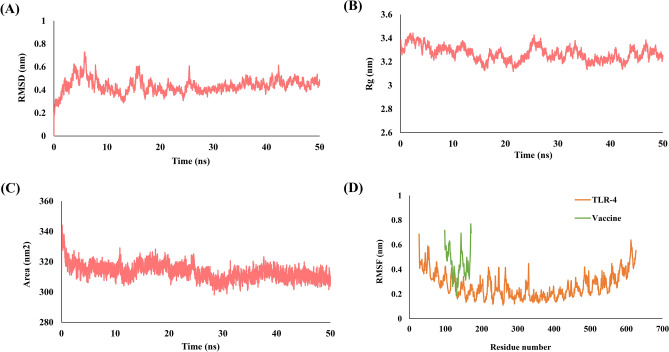
Representation of MD simulation analysis plot of proposed vaccine construct and TLR4 complex. (**A**) Root Mean Square Deviation (RMSD) of backbone atoms, (**B**) Radius of Gyration (R_g_), (**C**) Solvent Accessible Surface Area (SASA) analysis and (**D**) Root Mean Square Fluctuations (RMSF).

The molecular dynamic simulation was performed to examine the dynamics of stability and change in conformation of the vaccine construct and TLR4 complex. The complex was MD simulated for 50 ns long trajectory for better stability and conformation dynamics analysis. During the simulation receptor-ligand complex interaction and free binding energy were analysed using RMSD, RMSF, Rg and SASA. RMSD is the measurement of protein variability and provides information about protein stability. During the simulations, there was no significant fluctuation in the RMSD plot indicating receptor-ligand complex stability. The RMSD graph was found to be consistent at < 0.44 nm throughout the 50 ns simulation (Fig. 5A). We also performed RMSF analysis to conclude the flexibility of each amino acid residue in the receptor-ligand complex. A maximum RMSF of 0.68 nm and 0.76 nm was observed for the vaccine construct and TLR4 respectively, which is noteworthy and shows that residues in both receptor ligand complex are highly flexible (Fig. 5D). SASA is the measure of the surface area accessible to solvent molecules. The SASA value for our receptor-ligand complex was found to be consistent at an average of 313.41 nm^2^, suggesting the complex stability in the solvent environment (Fig. 5C). The radius of gyration for the receptor-ligand complex was also computed to be consistent at around 3.26 nm indicating that the complex is highly compact and will be stable in its biological environment (Fig. 5B). The vaccine construct and TLR4 complex after MD simulation can be seen in Fig. 4B.

### Codon optimization and in-silico cloning

**Figure 6:**
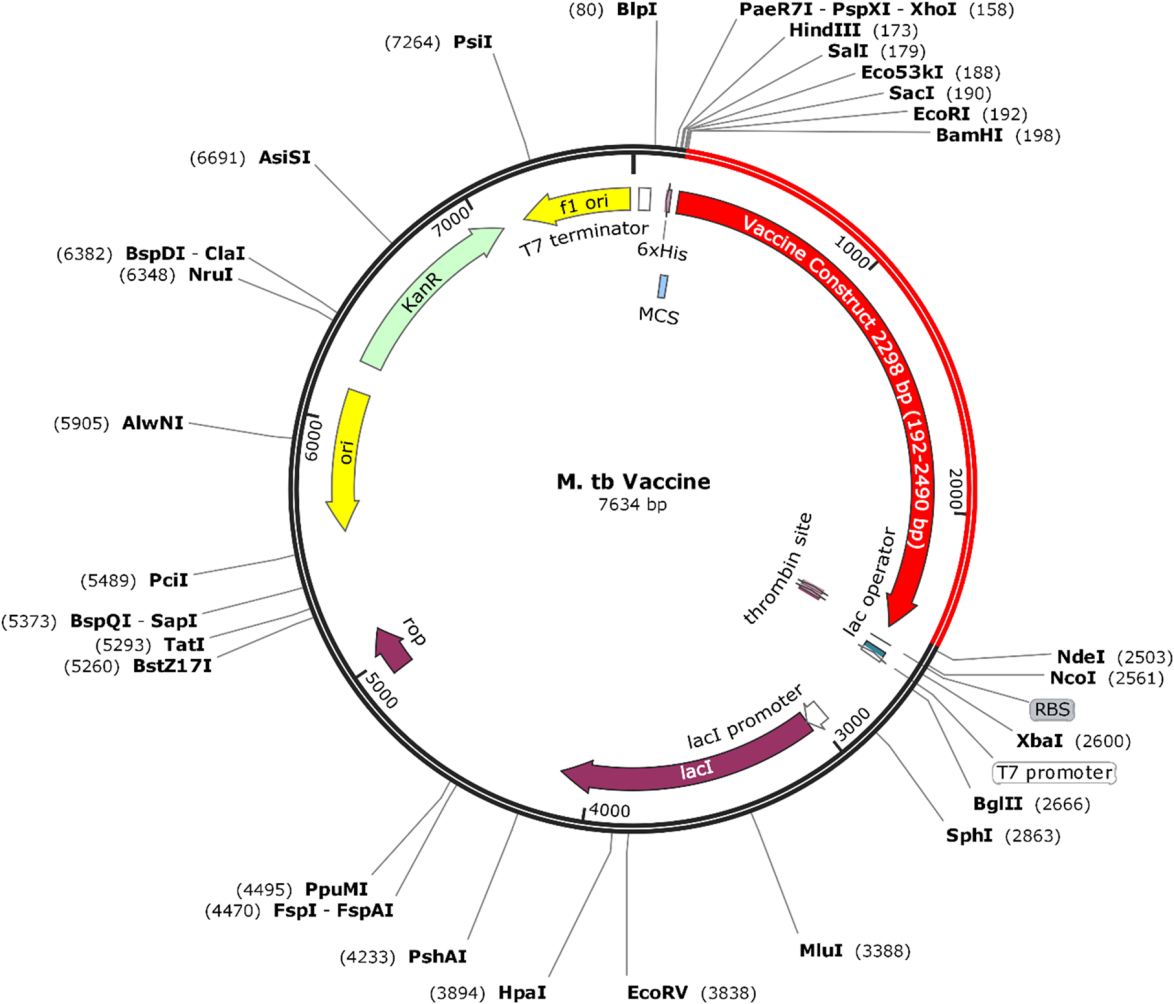
Optimized vaccine construct nucleotide sequence cloned inside pET28a(+) vector to ensure its expression in *E.coli* host. The gene is flanked with BamHI and NdeI restriction sites.*In-silico* cloning was performed using the free trial version of SnapGene tool (snapgene.com).

Codon optimization to ensure vaccine expression inside the *E. coli* (strain K12) host was performed using the JCat web server. The length of the optimized gene was calculated to be 2298 nucleotides with a CAI value of 0.99 and GC content of the optimized sequence was 55.04 percent. The values of CAI and percent, GC content is within the required range as described in methods. The optimized vaccine nucleotide sequence was flanked with BamHI and NdeI restriction sites and cloned inside the pET-28a( +) vector to ensure its optimized expression inside an *E. coli* host (Fig. 6).

### Immune simulation

**Figure 7:**
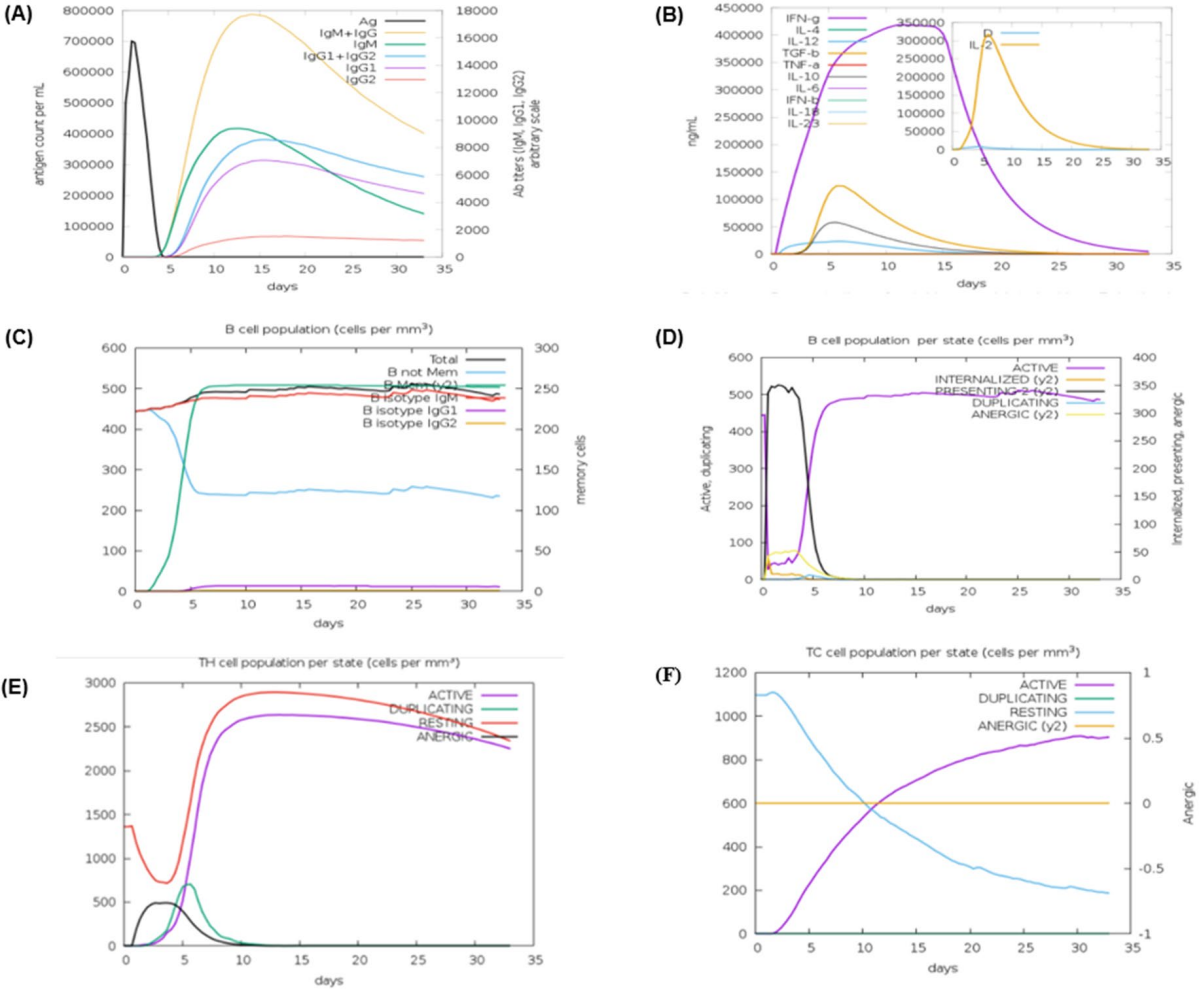
In silico immune simulation results using C-ImmSim web server (https://www.iac.cnr.it/~filippo/projects/c-immsim-online.html). (**A**) Antibodies produced in response to antigen exposure. (**B**) High level of IFN-γ production in response to antigens. (**C**) The rapid proliferation of B-cell along with memory B-cells. (**D**) Increased B-cell (active) production after a lag phase of 5–7 days after exposure to the vaccine chimera. (**E**) Increased T helper cell population and (**F**) Evolution of cytotoxic T-cells.

Immune simulation results yielded by the C-ImmSim server were consistent with a powerful immune reaction. As depicted from the graphs our vaccine construct mounts strong primary and secondary immune response. The primary immune response is characterized by the elevated level of the IgM antibodies after a lag period of 5–7 seven days of antigen exposure. The secondary immune response is characterized by the increased proliferation of B-cell as well as increased expression of IgM, IgG1 + IgG2 and IgG + IgM antibodies. The proposed vaccine construct not only elicits strong B-cell proliferation but also leads to the generation of memory B-cell. The vaccine construct also elicits a strong helper and cytotoxic T-cell immune response while it also marks an increased level of IFN-γ for a long duration of time (Fig. 7).

## Discussion

*Mycobacterium tuberculosis* (*M. tb*) is the greatest killer of the world population and is well known to actively tackle antibiotic treatment. The struggle of the scientific community for a single treatment of tuberculosis voices up the global need for tuberculosis treatment. While there are several anti-tuberculosis drugs available in the market, but they also seem to be ineffective against TB, as the pathogen has acquired a great potential to develop drug resistance in a less significant amount of time. In light of ever-expanding drug resistance and adverse effects associated with anti-TB drugs such as ototoxicity, hepatotoxicity, neuropsychiatricevents, hyperuricemia, gastrointestinal disturbance, vision loss, skin pigmentation etc^52^, a safe and efficacious vaccine could be an imperative arsenal against this deadly disease. At present, BCG is the only licensed anti-tuberculosis vaccine available in the market which is struggling with its variable global efficacy. The history of the BCG vaccine is nearly a century old which was first medically administered in 1921^53^. It is effective in mounting the protection against disseminated tuberculosis in young children but shows variable efficacy against pulmonary tuberculosis in adults^54^. The variations in the efficacy of the BCG can be attributed to the different protocols followed by several laboratories over the last few decades for BCG culture, which eventually gave rise to genetic variation among BCG strains^5^. The utilization of different media may also result in a variation to BCG efficacy, as there are shreds of evidences that proves that BCG cultured in Sauton media have better immunogenicity than the BCG cultured in Middlebrook 7H9 medium^55^. Another legitimising fact is the interference by non-tuberculous mycobacteria (NTM), as the studies show that prior exposure to NTM can mask the effect of BCG by inhibiting its replication^56^. Currently, there are several anti-tuberculosis vaccines that are in clinical trials, of which three vaccines viz. VMP1002, MIP and *M. vaccae* have made their way to phase III trials. These vaccines will be used as a booster for the BCG, indicating the variable efficacy of the vaccine. The introduction of a new traditional vaccine is extremely challenging as it requires a lot of laboratory investigations before several efficacy and safety trials, which demand a lot of time and can cost a large amount of money^57^. In recent years, immunoinformatic has opened the doors for drugs and vaccine development at a lightning-fast speed as compared to the traditional in vitro and in vivo approaches. Recently few immunoinformatic approaches have shown convincing results in various in vitro and in vivo experimental studies^58^. In a recent study, five rickettsia antigens were identified using the immunoinformatic and reverse vaccinology approach which during in vivo experiments protects against several types of rickettsia^59^. The immunoinformatic approach has been used several times to design a vaccine against many pathogens which are in the late phase of their clinical trials like against *Bacillus anthracis*, *Staphylococcus aureus*, *Bordetella pertussis*, *Plasmodium falciparum*, *Salmonella species*, *Candida albicans* and *Streptococcus canis*^58^. While considering the global crisis for effective tuberculosis treatment we have designed a multi-epitope vaccine using the experimentally proven immunogenic exosome vesicles-based antigens. Each of these antigenic proteins were analysed for their capacity to elicit the humoral, innate and cell-mediated immune response through predicting their B-cell, HTL and CTL epitopes, respectively. To ensure that the selected epitopes are antigenic enough to elicit the desired immune response while remaining non-allergenic and non-toxic to vaccine recipients these were evaluated for the same. All selected epitopes were merged using the appropriate linkers and to magnify the immune response of the vaccine a TLR4 agonist RpfE peptide was added to the N terminal of the construct which served as an adjuvant. Both TLR2 and TLR4 are involved in *M. tb* recognition during the infection as they activate macrophages and dendritic cell and thus influence the innate and adaptive immune system^60^. Since both TLR2 and TLR4 can be used as a receptor for the vaccine construct but it is known that low survival rate and high bacterial burden are observed in TLR4 mutant mice when infected with *M. tb*^61^ and most of our proteins are lipoproteins (LPS) and heat shock proteins (hsp70) so we have selected TLR4 immune receptor to be exploited for our study, as LPS and hsp70 are best recognized by TLR4 receptor^62^.Five vaccine constructs were designed, and homology modelled. The best 3D model of vaccine construct was refined and docked with the TLR4 monomer with best antigenicity score as well. The docked vaccine-TLR4 complex was subjected to molecular dynamic simulation to assess the stability of the macromolecule complex in their natural biological environment. The docked vaccine-TLR4 complex showed significant docking energies and was found to be stable. To validate the immunogenicity of the vaccine construct in serological analysis the vaccine construct should be expressed in its recombinant form in a suitable host. Therefore, the vaccine construct was codon optimised and *in-silico* cloned using pET28a( +) vector. Optimised vaccine construct sequence showed favourable codon adaptability index and GC content ensuring improved expression. Finally, an immune simulation experiment mimicking the innate network of the immune system involving B cells and T cells was carried out to assess humoral and cellular responses against the *M. tb* multi-epitope vaccine. The results of this investigation suggest a powerful immune reaction on the administration of the proposed vaccine construct.

In conclusion, our study demonstrates that the proposed vaccine candidate displays virtuous structural, desirable physiochemical and appealing immunological attributes that can generate a strong humoral and cellular immune response and therefore should be thrusted forward as a potential lead candidate for in vitro and in vivo evaluations against *M. tb*. Immune simulation results show that our vaccine construct can elicit the immune response consistent with our hypothesis. The proposed vaccine construct should be pushed for validation using in vitro binding studies exploiting purified TLR4 and the recombinant vaccine candidate followed by various serological assays to conclude the elicitation of a desired immune response.

## Supplementary Information

Supplementary Information.

## References

[1] World Health Organization (2019). Global tuberculosis report.

[2] Nagpal P (2020). Long-range replica exchange molecular dynamics guided drug repurposing against tyrosine kinase PtkA of *Mycobacterium tuberculosis*. Sci. Rep..

[3] Pablos-Mendez A, Gowda DK, Frieden TR (2002). Controlling multidrug-resistant tuberculosis and access to expensive drugs: a rational framework. Bull. World Health Organ..

[4] Brosch R (2007). Genome plasticity of BCG and impact on vaccine efficacy. Proc. Natl. Acad. Sci..

[5] Mangtani P (2014). Protection by BCG vaccine against tuberculosis: a systematic review of randomized controlled trials. Clin. Infect. Dis..

[6] Faust L, Schreiber Y, Bocking N (2019). A systematic review of BCG vaccination policies among high-risk groups in low TB-burden countries: implications for vaccination strategy in Canadian indigenous communities. BMC Public Health.

[7] Tkach M, Théry C (2016). Communication by extracellular vesicles: where we are and where we need to go. Cell.

[8] Lee J (2015). Proteomic analysis of extracellular vesicles derived from *Mycobacterium tuberculosis*. Proteomics.

[9] Schorey JS, Cheng Y, Singh PP, Smith VL (2015). Exosomes and other extracellular vesicles in host–pathogen interactions. EMBO Rep..

[10] Schorey JS, Harding CV (2016). Extracellular vesicles and infectious diseases: new complexity to an old story. J. Clin. Investig..

[11] Hart PD, Young MR, Gordon AH, Sullivan KH (1987). Inhibition of phagosome-lysosome fusion in macrophages by certain mycobacteria can be explained by inhibition of lysosomal movements observed after phagocytosis. J. Exp. Med..

[12] Xu S (1994). Intracellular trafficking in *Mycobacterium tuberculosis* and *Mycobacterium avium*-infected macrophages. J. Immunol..

[13] Mehaffy C, Dobos KM, Nahid P, Kruh-Garcia NA (2017). Second generation multiple reaction monitoring assays for enhanced detection of ultra-low abundance *Mycobacterium tuberculosis* peptides in human serum. Clin. Proteomics.

[14] Chuang Y-M, Pinn ML, Karakousis PC, Hung C-F (2018). Intranasal Immunization with DnaK protein induces protective mucosal immunity against tuberculosis in CD4-depleted mice. Front. Cell. Infect. Microbiol..

[15] Ferraz JC (2004). A heterologous DNA priming-*Mycobacterium bovis* BCG boosting immunization strategy using mycobacterial Hsp70, Hsp65, and Apa antigens improves protection against tuberculosis in mice. Infect. Immun..

[16] Bandyopadhyay B, Das Gupta T, Roy D, Das Gupta SK (2012). DnaK dependence of the mycobacterial stress-responsive regulator HspR is mediated through its hydrophobic C-terminal tail. J. Bacteriol..

[17] Kim WS (2018). *Mycobacterium tuberculosis* GrpE, a heat-shock stress responsive chaperone, promotes Th1-biased T cell immune response via TLR4-mediated activation of dendritic cells. Front. Cell. Infect. Microbiol..

[18] Griffin JE (2011). High-resolution phenotypic profiling defines genes essential for mycobacterial growth and cholesterol catabolism. PLOS Pathog..

[19] Sánchez A, Espinosa P, García T, Mancilla R (2012). The 19 kDa *Mycobacterium tuberculosis* lipoprotein (LpqH) induces macrophage apoptosis through extrinsic and intrinsic pathways: a role for the mitochondrial apoptosis-inducing factor. Clin. Dev. Immunol..

[20] Hougardy J-M (2007). Heparin-binding-hemagglutinin-induced IFN-γ release as a diagnostic tool for latent tuberculosis. PLoS ONE.

[21] Bigi F (2000). The gene encoding P27 lipoprotein and a putative antibiotic-resistance gene form an operon in *Mycobacterium tuberculosis* and *Mycobacterium bovis*. Microbiology.

[22] Drage MG (2010). *Mycobacterium tuberculosis* lipoprotein LprG (Rv1411c) binds triacylated glycolipid agonists of Toll-like receptor 2. Nat. Struct. Mol. Biol..

[23] Martinot AJ (2016). Mycobacterial metabolic syndrome: LprG and Rv1410 regulate triacylglyceride levels, growth rate and virulence in *Mycobacterium tuberculosis*. PLoS Pathog..

[24] Bianco MV (2011). Role of P27–P55 operon from *Mycobacterium tuberculosis* in the resistance to toxic compounds. BMC Infect. Dis..

[25] Wang L (2017). *Mycobacterium tuberculosis* lipoprotein MPT83 induces apoptosis of infected macrophages by activating the TLR2/p38/COX-2 signaling pathway. J. Immunol..

[26] NCBI Resource Coordinators. Database resources of the National Center for Biotechnology Information. *Nucleic Acids Res. 46*, D8–D13 (2018). 10.1093/nar/gkx1095 PMC5753372 29140470

[27] Choi H-G (2015). *Mycobacterium tuberculosis* RpfE promotes simultaneous Th1- and Th17-type T-cell immunity via TLR4-dependent maturation of dendritic cells. Eur. J. Immunol..

[28] Jung ID (2011). Enhanced efficacy of therapeutic cancer vaccines produced by co-treatment with *Mycobacterium tuberculosis* heparin-binding hemagglutinin, a novel TLR4 agonist. Cancer Res..

[29] Lee SJ (2014). A potential protein adjuvant derived from *Mycobacterium tuberculosis* Rv0652 enhances dendritic cells-based tumor immunotherapy. PLoS ONE.

[30] Saha S, Raghava GPS (2006). Prediction of continuous B-cell epitopes in an antigen using recurrent neural network. Proteins Struct. Funct. Bioinform..

[31] Fleri W (2017). The immune epitope database and analysis resource in epitope discovery and synthetic vaccine design. Front. Immunol..

[32] Doytchinova IA, Flower DR (2007). VaxiJen: a server for prediction of protective antigens, tumour antigens and subunit vaccines. BMC Bioinform..

[33] Dimitrov I, Flower DR, Doytchinova I (2013). AllerTOP—a server for in silico prediction of allergens. BMC Bioinform..

[34] Gupta S (2013). In silico approach for predicting toxicity of peptides and proteins. PLoS ONE.

[35] Roy A, Kucukural A, Zhang Y (2010). I-TASSER: a unified platform for automated protein structure and function prediction. Nat. Protoc..

[36] Waterhouse A (2018). SWISS-MODEL: homology modelling of protein structures and complexes. Nucleic Acids Res..

[37] Kelley LA, Mezulis S, Yates CM, Wass MN, Sternberg MJE (2015). The Phyre2 web portal for protein modeling, prediction and analysis. Nat. Protoc..

[38] Bhattacharya A, Tejero R, Montelione GT (2007). Evaluating protein structures determined by structural genomics consortia. Proteins Struct. Funct. Bioinform..

[39] Gasteiger, E. *et al.* Protein identification and analysis tools on the ExPASy server. in *The Proteomics Protocols Handbook* (ed. Walker, J. M.) (Humana Press, 2005).

[40] Heo L, Park H, Seok C (2013). GalaxyRefine: protein structure refinement driven by side-chain repacking. Nucleic Acids Res..

[41] Berman HM (2000). The protein data bank. Nucleic Acids Res..

[42] Park BS (2009). The structural basis of lipopolysaccharide recognition by the TLR4–MD-2 complex. Nature.

[43] Trott O, Olson AJ (2010). AutoDock Vina: improving the speed and accuracy of docking with a new scoring function, efficient optimization, and multithreading. J. Comput. Chem..

[44] Tian W, Chen C, Lei X, Zhao J, Liang J (2018). CASTp 3.0: computed atlas of surface topography of proteins. Nucleic Acids Res..

[45] de Vries SJ, van Dijk M, Bonvin AMJJ (2010). The HADDOCK web server for data-driven biomolecular docking. Nat. Protoc..

[46] Van Der Spoel D (2005). GROMACS: fast, flexible, and free. J. Comput. Chem..

[47] Grote A (2005). JCat: a novel tool to adapt codon usage of a target gene to its potential expression host. Nucleic Acids Res..

[48] Morla S, Makhija A, Kumar S (2016). Synonymous codon usage pattern in glycoprotein gene of rabies virus. Gene.

[49] Ali M (2017). Exploring dengue genome to construct a multi-epitope based subunit vaccine by utilizing immunoinformatics approach to battle against dengue infection. Sci. Rep..

[50] Rapin N, Lund O, Bernaschi M, Castiglione F (2010). Computational immunology meets bioinformatics: the use of prediction tools for molecular binding in the simulation of the immune system. PLoS ONE.

[51] Liu H, Irvine DJ (2015). Guiding principles in the design of molecular bioconjugates for vaccine applications. Bioconj. Chem..

[52] Gülbay BE (2006). Side effects due to primary antituberculosis drugs during the initial phase of therapy in 1149 hospitalized patients for tuberculosis. Respir. Med..

[53] World Health Organization. BCG vaccine: WHO position paper, February 2018 - Recommendations. *Vaccine* **36**, 3408–3410 (2018). 10.1016/j.vaccine.2018.03.009 29609965

[54] Trunz BB, Fine PEM, Dye C (2006). Effect of BCG vaccination on childhood tuberculous meningitis and miliary tuberculosis worldwide: a meta-analysis and assessment of cost-effectiveness. Lancet.

[55] Venkataswamy MM (2012). In vitro culture medium influences the vaccine efficacy of *Mycobacterium bovis* BCG. Vaccine.

[56] Brandt L (2002). Failure of the *Mycobacterium bovis* BCG vaccine: some species of environmental mycobacteria block multiplication of bcg and induction of protective immunity to tuberculosis. Infect. Immun..

[57] Martin C, Aguilo N, Marinova D, Gonzalo-Asensio J (2020). Update on TB vaccine pipeline. Appl. Sci..

[58] Mehla K, Ramana J (2016). Identification of epitope-based peptide vaccine candidates against enterotoxigenic *Escherichia coli*: a comparative genomics and immunoinformatics approach. Mol. Biosyst..

[59] Caro-Gomez E, Gazi M, Goez Y, Valbuena G (2014). Discovery of novel cross-protective *Rickettsia prowazekii* T-cell antigens using a combined reverse vaccinology and in vivo screening approach. Vaccine.

[60] Carmona J (2013). *Mycobacterium tuberculosis* strains are differentially recognized by TLRs with an impact on the immune response. PLoS ONE.

[61] Branger J (2004). Toll-like receptor 4 plays a protective role in pulmonary tuberculosis in mice. Int. Immunol..

[62] Casella CR, Mitchell TC (2008). Putting endotoxin to work for us: monophosphoryl lipid A as a safe and effective vaccine adjuvant. Cell. Mol. Life Sci..

