# Author Correction: An immunoinformatics approach to design a multi-epitope vaccine against *Mycobacterium tuberculosis* exploiting secreted exosome proteins

**DOI:** 10.1038/s41598-021-96314-7

**Published:** 2021-08-13

**Authors:** Rahul Sharma, Vikrant Singh Rajput, Salma Jamal, Abhinav Grover, Sonam Grover

**Affiliations:** 1 Institute of Molecular Medicine, Jamia Hamdard, New Delhi, 110062 India; 2 School of Biotechnology, Jawaharlal Nehru University, New Delhi, 110067 India

Correction to: *Scientific Reports* https://doi.org/10.1038/s41598-021-93266-w, published online 05 July 2021

In the original version of this Article, author Salma Jamal was omitted as a corresponding author. Correspondence and requests for material should also be addressed to salmajamal@jamiahamdard.ac.in.

The original Article and accompanying Supplementary Information file have been corrected.

