# Supplementary material for: An immunoinformatics approach to design a multi-epitope vaccine against Mycobacterium tuberculosis exploiting secreted exosome proteins
Source: Sci Rep. 2021 Jul 5;11:13836. doi: 10.1038/s41598-021-93266-w (PMC8257786; doi:10.1038/s41598-021-93266-w)
Supplement: Supplementary file 1 — Supplementary Information. [file 41598_2021_93266_MOESM1_ESM.docx]

**An Immunoinformatics approach to design a multi-epitope vaccine against Mycobacterium tuberculosis exploiting secreted exosome proteins.**

Rahul Sharma1#, Vikrant Singh Rajput2#, Salma Jamal1, Abhinav Grover2* and Sonam Grover1*

# These authors contributed equally to the study.

1Institute of Molecular Medicine, Jamia Hamdard, 110062, India

2School of Biotechnology, Jawahar Lal Nehru University, 110067, India

**Supplementary Information**

**Table1- Selected B-cell (green), HTL (blue) and CTL (magenta) epitopes. Numeric digits in brackets represents the starts position of the epitope in the respective protein.**

| Gene Name | B-cell epitope | CTL epitope | HTL epitope |
| --- | --- | --- | --- |
| DnaK | EAHAEEDRKRREEADV(494) | GEVKDVLLL (358) | EVKDVLLLDVTPLSL (359) |
|  |  | DAVITTPAY (113) |  |
|  |  | RTTPSIVAF (34) |  |
| GrpE | DGTAVADTAENDQADQ (202) |  |  |
|  | EGEDFDPVLHEAVQHE (138) |  |  |
| LpqH | LGYTSGTGQGNASATK (110) | ATKDGSHYK (123) | LTVAVAGAAILVAGL (5) |
|  | PGAASGPKVVIDGKDQ (44) | AVAGAAILV (8) | NVNIAIGGAATGIAA (73) |
|  |  | VLTDGNPPEV (88) |  |
|  |  |  |  |
|  |  |  |  |
|  |  |  |  |
| HbhA | TDTRSRVEESRARLTK (44) | EEVSARAEGY (116) |  |
|  | AKKAPAKKAAAKKVTQ (183) | QSFEEVSAR (113) |  |
|  | APAKKAAPAKKAAAKK (170) | AAAKKVTQK (191) |  |
| LprA | GGCSTEGDAGKASDTA (23) | APTKDTSVTL (220) | CSVVAAATAILAVVL (6) |
|  | LVQIQIAPTKDTSVTL (214) |  | DGSSHLVQIQIAPTK (209) |
|  | PNLRVTKLEGDISNTP (74) |  |  |
|  | VRLAVTGDVPNLRVTK (65) |  |  |
|  |  |  |  |
|  |  |  |  |
| LprG | PGLSLKTLSGDLTTNP (69) |  | AVLAAVSIAATVVAG (12) |
| Mpt83 | ADLIGRGCAQYAAQNP (57) |  | IGARDDLMVNNAGLV (183) |
|  | DTSPKPATSPAAPVTT (34) |  | KPAAAASLAAIAIAF (6) |
|  | QYAAQNPTGPGSVAGM (66) |  | AAAASLAAIAIAFLA (130) |
|  |  |  |  |
|  |  |  |  |
|  |  |  |  |
|  |  |  |  |

**Table2- Vaccine-TLR4 docking score**

| HADDOCK score | -83.1 +/- 4.5 |
| --- | --- |
| Cluster size | 117 |
| RMSD from the overall lowest-energy structure | 0.8 +/- 0.5 |
| Van der Waals energy | -47.5 +/- 3.5 |
| Electrostatic energy | -168.1 +/- 18.3 |
| Desolvation energy | -2.3 +/- 4.1 |
| Restraints violation energy | 3.1 +/- 0.75 |
| Buried Surface Area | 1407.6 +/- 77.2 |
| Z-Score | -1.2 |

**Supplementary Figure**

MKNARTTLIAAAIAGTLVTRSPAGIANADDAGLDPNAAAGPDAVGFDPNLPPAPDAAPVDTPPAPEDAGFDPNLPPPLAPDFLSPPAEEAPPVPVAYSVNWDAIAQCESGGNWSINTGNGYYGGLQFTAGTWRANGGSGSAANASREEQIRVAENVLRSQGIRAWPVCGRRGEAAAKEAHAEEDRKRREEADVKKDGTAVADTAENDQADQKKEGEDFDPVLHEAVQHEKKLGYTSGTGQGNASATKKKPGAASGPKVVIDGKDQKKTDTRSRVEESRARLTKKKAKKAPAKKAAAKKVTQKKAPAKKAAPAKKAAAKKKKGGCSTEGDAGKASDTAKKLVQIQIAPTKDTSVTLKKPNLRVTKLEGDISNTPKKVRLAVTGDVPNLRVTKKKPGLSLKTLSGDLTTNPKKADLIGRGCAQYAAQNPKKDTSPKPATSPAAPVTTKKQYAAQNPTGPGSVAGMGPGPGEVKDVLLLDVTPLSLGPGPGLTVAVAGAAILVAGLGPGPGNVNIAIGGAATGIAAGPGPGCSVVAAATAILAVVLGPGPGDGSSHLVQIQIAPTKGPGPGAVLAAVSIAATVVAGGPGPGIGARDDLMVNNAGLVGPGPGKPAAAASLAAIAIAFGPGPGAAAASLAAIAIAFLAAAYGEVKDVLLLAAYDAVITTPAYAAYRTTPSIVAFAAYATKDGSHYKAAYAVAGAAILVAAYVLTDGNPPEVAAYEEVSARAEGYAAYQSFEEVSARAAYAAAKKVTQKAAYAPTKDTSVTL

**Supplementary figure 1-** The engineered vaccine construct sequence. The vaccine candidate is designed in the sequential manner which is- Adjuvant-B-cell-HTL-CTL. In the vaccine sequence, the first 172 amino acids are occupied by an adjuvant (Black colour), next 5 amino acids are of EAAAK linker (Purple colour) and then from number 178-463 amino acids constitute the B-cell epitopes (Green colour) whereas 464-643 and 644-766correspond to HTL (Magenta colour) and CTL epitopes (Bluecolour), respectively. All the linkers, except EAKKKare represented in red colour.
